# Anemia, hypoalbuminemia, and elevated troponin levels as risk factors for respiratory failure in patients with severe exacerbations of chronic obstructive pulmonary disease requiring invasive mechanical ventilation

**DOI:** 10.3325/cmj.2017.58.395

**Published:** 2017-12

**Authors:** Gordana Pavliša, Marina Labor, Hrvoje Puretić, Ana Hećimović, Marko Jakopović, Miroslav Samaržija

**Affiliations:** 1 Clinical Center for Pulmonary Diseases “Jordanovac”, University Hospital Center Zagreb, Zagreb, Croatia; 2 School of Medicine, University of Zagreb, Zagreb, Croatia; 3 Department of Pulmonology, University Hospital Center Osijek, Osijek, Croatia; 4 Faculty of Medicine, J.J. Strossmayer University of Osijek, Osijek, Croatia; * The first two authors contributed equally.

## Abstract

**Aim:**

To determine in-hospital and post-discharge mortality, readmission rates, and predictors of invasive mechanical ventilation (IMV) in patients treated at intensive care unit (ICU) due to acute exacerbations of chronic obstructive pulmonary disease (AECOPD).

**Methods:**

A retrospective observational cohort study included all patients treated at a respiratory ICU for AECOPD during one year. A total of 62 patients (41 men) with mean age 68.4 ± 10.4 years were analyzed for outcomes including in-hospital and post-discharge mortality, readmission rates, and IMV. Patients’ demographic, hematologic, biochemical data and arterial blood gas (ABG) values were recorded on admission to hospital. Mean duration of follow-up time was 2.4 years.

**Results:**

Of 62 patients, 7 (11.3%) died during incident hospitalization and 21 (33.9%) died during the follow-up. The overall 2.4-year mortality was 45.2%. Twenty nine (46.8%) patients were readmitted due to AECOPD. The average number of readmissions was 1.2. Multivariate analysis showed that blood pH, bicarbonate levels, low albumin, low serum chloride, and low hemoglobin were significant predictors of IMV during incident hospitalization (*P* < 0.001 for the overall model fit).

**Conclusion:**

High in-hospital and post-discharge mortality and high readmission rates in our patients treated due to AECOPD at ICU indicate that these patients represent a high risk group in need of close monitoring. Our results suggested that anemia, hypoalbuminemia, and elevated troponin levels were risk factors for the need of IMV in severe AECOPD. Identification of such high-risk patients could provide the opportunity for administration of an appropriate and timely treatment.

Chronic obstructive pulmonary disease (COPD) is a major cause of morbidity and mortality affecting approximately 10% of the total adult population (1,2). The natural course of the disease is disrupted by exacerbations, representing a major event for COPD management due to its negative impact on disease progression, health status, and quality of life (1,3,4). About 10% of all hospitalizations are directly or indirectly related to COPD, which places a significant burden on the health care system (5). Acute COPD exacerbations (AECOPD) often result in worsening of respiratory failure or incident one. AECOPD are a common cause of intensive care unit (ICU) admissions of patients requiring non-invasive (NIV) or invasive mechanical ventilation (IMV). Long-term mortality of in-hospital COPD patients treated for AECOPD is high, with a survival rate comparable to that of a lung cancer (6,7). According to Intensive Care National Audit & Research Centre (ICNARC) Case Mix Program Database, 23.1% of patients die during their stay at the ICU, and 38.8% during hospitalization (8). No data are available for Croatia. The ICU management of these high-risk patients includes ventilator support (NIV or IMV), inhaled bronchodilators, systemic corticosteroids, and antibiotics, but the evidence behind these interventions almost inadequate (9).

NIV represents an effective method for the management of hypercapnic respiratory failure in AECOPD. For the management of AECOPD, apart from ICUs, NIV is today frequently applied in regular wards. However, NIV failure and the need for IMV can be expected in about a quarter of patients (10). Therefore, when NIV is used, it is necessary to monitor patients carefully. Identification of risk factors for NIV failure could help define a group of patients who require more stringent monitoring or additional intervention. This could allow for targeted interventions that could improve survival, directing medical resources cost-effectively.

The aim of our study was to determine in-hospital and post-discharge mortality, predictors of survival time, readmission rates, and NIV failure rates in COPD patients treated at ICU due to a high degree of respiratory failure and clinical severity of AECOPD. This study was a hypothesis-generating association study for the possible predictors of poor outcomes, such as in-hospital and post discharge mortality, readmissions, and NIV failure, in this group of high-risk COPD patients, representing Level 4 of evidence (11).

## METHODS

### Study design

This was a retrospective observational cohort study of all patients treated at our ICU for AECOPD during one year, with the follow-up for readmissions and post-discharge mortality. We analyzed variables routinely collected during the treatment of AECOPD patients, ie, universal and easily accessible parameters. As this was a retrospective analysis of anonymized clinically obtained data, there was no need for patients to sign the informed consent. The competent Ethics Committee approved that in specific cases, where there was no data in hospital database about the death of a patient through the follow-up period, the patient or a family member would be contacted by phone just for this specific information (time of death, if any). Verbal consent was obtained at the time of interview before obtaining the information.

### Patient selection

We included all 62 patients treated for AECOPD and subsequent respiratory failure at our respiratory ICU from September 2013 through September 2014. All patients included in this study had already been diagnosed with COPD according to Global Initiative for Chronic Obstructive Lung Disease (GOLD) by their physician (1). AECOPD was defined as a worsening in at least two of the following symptoms: cough, purulent sputum, and dyspnea (1). Patients were admitted to the ICU due to one or more of the following indications: persistent or worsening hypoxemia or severe or worsening hypercapnia despite the initial treatment, respiratory acidosis, or impaired consciousness (confusion, lethargy, or coma) due to hypercapnia. To avoid selection bias, we included all patients consecutively treated under this diagnosis. Patient data collected on admission included demographic data (age, sex), duration of COPD, body mass index (BMI, calculated as body mass in kilograms divided by squared body height in meters), and comorbidities (pneumonia, hypertension, coronary heart disease, pulmonary hypertension, chronic pulmonary heart, cardiac decompensation, diabetes, obstructive sleep apnea, psychiatric disorders, and malignancy). The duration of COPD was defined from the date when the patient first contacted a physician due to the problems with chronic cough, sputum, and breathlessness.

### Study outcomes

The primary outcomes were in-hospital mortality and death during the follow-up period. As this was a retrospective study, only the date of death was available for all deceased patients. Data on the cause of death were not reliable and accurate because, in most cases (even in-hospital deaths), autopsy was not performed because it is not mandatory. The secondary outcomes were the need for readmission due to AECOPD during the follow-up period, the time to first readmission, and the need for IMV during incident hospitalization.

Data on the IMV and in-hospital mortality were obtained from patients’ hospital medical records. Mortality data after incident hospitalization and readmission data were obtained through telephone interviews with patients or members of their family or from hospital database.

Data on the predictors of study outcomes were also obtained from patients’ hospital medical records, including demographic and clinical data and values of hematologic and biochemical parameters and arterial blood gases. The average follow-up period was 2.4 years.

### Laboratory analyses

All analyzed data were extracted from patients’ hospital medical records. According to the in-house protocol, 2 blood samples were taken at the time of admission. The first sample was arterial blood taken for blood gases analysis, which was performed on ABL800 Flex (Radiometer, Copenhagen, Denmark) (12). The second sample was venous blood taken for the analysis of the following parameters: hematologic parameters, prothrombin time, activated partial thromboplastin time, fibrinogen, D-dimer, glucose, urea, creatinine, bilirubin, aspartate transaminase, alanine transaminase, gamma glutamyl transferase, alkaline phosphatase, creatine kinase, lactate dehydrogenase, sodium, potassium, chloride, troponin T, total iron, unsaturated iron-binding capacity, total iron-binding capacity, ferritin, total serum protein, albumin, and protein electrophoresis. Anemia was defined according to the World Health Organization criteria, ie, as the concentration of hemoglobin (Hgb)<13 g/dL in men and <12 g/dL in women (13).

### Treatment

Patients were treated according to GOLD recommendations for AECOPD (1). Patients were treated with 40 mg of intravenous methylprednisolone, nebulized bronchodilators (inhaled salbutamol and ipratropium bromide), and theophylline. Antibiotics were used in patients with signs of bacterial infection present. Comorbidities, such as arterial hypertension, coronary heart disease, cardiac decompensation, diabetes, and psychiatric disorders, were treated according to the principles of good clinical practice. Respiratory failure was managed with oxygen supplementation, NIV or IMV, according to the individual needs to achieve satisfactory arterial blood oxygen saturation of at least 88%-90% and to maintain pH within the physiological range. Patients were carefully monitored during treatment. NIV and IMV were also applied according to GOLD recommendations (1). NIV was used in patients with respiratory acidosis, severe dyspnea with clinical signs suggestive of respiratory muscle fatigue, increased work of breathing, and persistent hypoxemia despite supplemental oxygen therapy. In patients who were unable to tolerate NIV or in patients with NIV failure, IMV was initiated. Also, IMV was used in patients after cardiac or respiratory arrest, those unable to remove mucus secretion, those who had severe hemodynamic instability without response to fluids and vasoactive drugs, and patients with severe cardiac arrhythmias (1).

### Statistical analysis

Categorical variables were presented as numbers and proportions (%). Quantitative variables were presented as means and standard deviations (SD) or as medians with interquartile range (IQR), depending on the normality of distribution tested using Kolmogorov-Smirnov test. Differences between groups for categorical variables were evaluated using the χ^2^ test or Fisher exact test. Differences for quantitative variables were evaluated using Student’s *t* test or Mann-Whitney U-test depending on the distribution. Univariate regression analysis was performed to identify variables associated with outcome measures at *P* < 0.10. These variables were used to build multivariable models defining predictors and prediction models for outcome measures including the need for IMV, readmission during follow-up, time to next hospitalization, and survival time after incident hospitalization, using stepwise multivariable logistic regression analysis. Stepwise Cox proportional hazard regression was used for survival analysis. Statistical analyses were done using STATISTICA version 12 (StatSoft, Inc., OK, USA).

## RESULTS

The analyses included all 62 patients admitted to ICU for the treatment of severe AECOPD. Patients in need for IMV were of comparative age, sex, and body composition (Table 1). The duration of COPD was 3 years longer in IMV group of patients, but this difference did not reach statistical significance. Comparable distribution regarding comorbidities was found for both groups, with a comparable NIV duration between the groups, but with a significantly longer ICU stay in IMV group.

**Table 1:** Baseline characteristics of patients with acute exacerbation of chronic obstructive pulmonary disease in total and according to their need for mechanical ventilation*

Patient characteristics	No. (%) of patients†			*P*
	total (N = 62)	mechanical ventilation (n = 14)	no mechanical ventilation (n = 48)	
Age on admission (years)	68.4 ± 10.4	67.4 ± 11.2	68.6 ± 10.4	0.725
Women	21 (33.9)	6 (42.9)	15 (31.9)	0.449
BMI (kg/m^2^)	26.19 ± 6.04	24.30 ± 4.99	26.73 ± 6.25	0.205
Duration of COPD (years)	12.6 ± 5.5	14.7 ± 6.5	11.6 ± 5.0	0.277
Comorbidities				
pneumonia	33 (53.2)	7 (50.0)	26 (54.2)	0.726
heart failure	15 (24.2)	5 (35.7)	11 (22.9)	0.358
renal failure	12 (19.4)	3 (21.4)	9 (18.8)	0.851
diabetes mellitus	18 (29.0)	4 (28.6)	14 (29.2)	0.930
psychiatric disorder	15 (24.2)	3 (21.4)	12 (25.0)	0.101
malignancy	5 (8.1)	0 (0.0)	5 (10.4)	0.203
bronchiectasis	3 (4.8)	1 (7.1)	2 (4.2)	0.549
hypertension	21 (33.9)	3 (21.4)	18 (37.5)	0.244
obstructive sleep apnea	5 (8.1)	0 (0)	5 (10.4)	0.203
coronary artery disease	23 (37.1)	7 (50.0)	16 (33.3)	0.280
pulmonary heart disease	30 (48.4)	9 (64.3)	21 (43.8)	0.198
pulmonary hypertension	15 (24.2)	5 (35.7)	10 (20.8)	0.271
ICU stay (days)	7 (4 to 10)	10.5 (8 to 14)	6 (4 to 9)	0.003
Non-invasive ventilation (h)	22 (9 to 48)	15.5 (8 to 130)	22 (10 to 42)	0.783
Died	28 (45.2)	10 (71.4)	18 (37.5)	0.025
during incident hospitalization	7 (11.3)	4 (28.6)	3 (6.3)	0.020
during the follow-up time	21 (33.9)	6 (42.8)	15 (31.2)	0.419

*Abbreviations: SD – standard deviation, BMI – body mass index, COPD – chronic obstructive pulmonary disease, ICU – intensive care unit.

†Data are presented as numbers (%), medians with interquartile range (IQR), and mean with standard deviation (SD). Statistical significance for group comparisons was tested using χ^2^ or Fisher exact test for categorical variables, Mann-Whitney U test for quantitative variables presented as median (IQR), and Student’s *t* test for variables presented as mean±SD.

Patients receiving IMV had significantly lower red blood cell counts, hemoglobin, and hematocrit values and higher white blood cell counts, but their platelet, neutrophil, lymphocyte, and monocyte counts were not significantly higher (Table 2). There were no significant differences between the groups for coagulation factors, serum glucose, and renal and liver function markers, except for bilirubin, which was significantly lower in IMV group. Significantly lower values were found for chloride serum levels, albumin, A/G ratio, and significantly higher for troponin, and alpha1 globulin.

**Table 2:** Hematology, coagulation, and biochemistry parameters according to the need for mechanical ventilation (N = 62)

	Parameter values*			
Parameters	total (N = 62)	mechanical ventilation (n = 14)	no mechanical ventilation (n = 48)	*P*
Erythrocyte count (10^12^/L)	4.65 ± 0.84	4.04 ± 0.70	4.86 ± 0.78	0.001
Hemoglobin (g/L)	134.6 ± 21.9	116.9 ± 19.9	140.4 ± 19.5	<0.001
Hematocrit (L/L)	0.429 ± 0.073	0.370 ± 0.060	0.449 ± 0.066	<0.001
Platelet count (10^9^/L)	256 ± 112	306 ± 146	241 ± 98	0.080
Leukocyte count (10^9^/L)	11.5 ± 4.2	13.6 ± 4.4	11.0 ± 4.0	0.036
Neutrophils (10^9^/L)	9.4 ± 3.8	10.6 ± 4.0	9.0 ± 3.7	0.170
Lymphocytes (10^9^/L)	0.86 (0.50 to 1.20)	1.15 (0.5 to 1.5)	0.7 (0.42 to 1.10)	0.136
Monocytes (10^9^/L)	0.60 (0.30 to 0.98)	0.94 (0.3 to 1.1)	0.5 (0.3 to 0.8)	0.175
Prothrombin time	0.90 ± 0.27	0.92 ± 0.24	0.89 ± 0.28	0.706
Activated partial thromboplastin time (s)	26.0 ± 5.50	25.0 (23.8 to 27.4)	25.5 (23.0 to 29.0)	0.468
Fibrinogen (s)	5.21 ± 1.90	5.49 ± 1.68	5.13 ± 1.97	0.560
D dimer (μg/mL)	1.29 (0.76 to 2.35)	1.96 (1.09 to 4.35)	1.23 (0.61 to 1.99)	0.118
Blood glucose (mmol/L)	8.8 ± 3.6	9.1 ± 4.0	8.8 ± 3.5	0.747
Urea (mg/L)	8.4 (5.5 to 11.0)	8.1 (5.5 to 10.2)	8.4 (5.5 to 11.0)	0.764
Creatinine (mg/L)	99.5 (86.0 to 133.0)	97 (66 to 112)	104 (88 to 134)	0.143
Bilirubin (μmol/L)	7 (5 to 12)	5 (3 to 6)	7 (5 to 12)	0.011
Aspartate transaminase (U/L)	23 (18 to 35)	26 (17 to 35)	23 (18 to 33)	0.764
Alanine transaminase (U/L)	21 (13 to 29)	17.5 (14 to 28)	21 (12 to 30)	0.804
Gamma glutamyl transferase (U/L)	22.5 (15.5 to 46.0)	27 (19 to 64)	22 (14 to 38)	0.242
Alkaline phosphatase (U/L)	80.2 ± 42.8	93.4 ± 55.5	76.3 ± 38.1	0.194
Creatine kinase (U/L)	64 (37 to 92)	82 (47.5 to 131.5)	59.5 (33 to 90)	0.394
Lactate dehydrogenase (U/L)	190.5 (158 to 223)	202.5 (176 to 256)	184 (154.5 to 216.5)	0.151
Potassium (mmol/L)	4.5 ± 0.6	4.3 ± 0.5	4.5 ± 0.7	0.416
Sodium (mmol/L)	139 ± 5	137 ± 5	140 ± 4	0.063
Chloride (mmol/L)	94 ± 6	91 ± 7	95 ± 5	0.019
C reactive protein (mg/L)	40.2 (11.5 to 95.2)	60.75 (22.9 to 94.3)	34.1 (8.0 to 95.2)	0.261
Total serum protein (g/L)	64.0 ± 7.1	61.4 ± 8.8	64.8 ± 6.4	0.116
Albumin (g/L)	34.8 ± 6.4	30.4 ± 6.3	36.3 ± 5.8	0.002
Troponin T (ng/mL)	0.022 (0.017 to 0.054)	0.044 (0.022 to 0.131)	0.021 (0.014 to 0.034)	0.006
Sedimentation rate (mm/h)	38.7 ± 29.3	46 ± 29	36 ± 29	0.309
Anti-thrombin 3 (%)	78.9 ± 28.6	83.8 ± 34.4	77.5 ± 27.1	0.528
Total iron (µmol/L)	7 (5 to 11)	6 (5 to 10)	8.5 (5 to 11)	0.601
Unsaturated iron-binding capacity (µmol/L)	42 ± 16	38 ± 12	43 ± 16	0.359
Total iron-binding capacity (µmol/L)	51 ± 14	46 ± 11	52 ± 14	0.211
Ferritin (ng/mL)	89.1 (27.8 to 358.2)	238.5 (60.0 to 360.2)	59.4 (26.6 to 343.0)	0.363
Alpha-1 globulin (g/L)	4.30 ± 1.03	4.9 ± 0.9	4.1 ± 1.0	0.018
Alpha-2 globulin (g/L)	8.30 ± 1.86	8.2 ± 1.7	8.3 ± 1.9	0.909
Beta globulin (g/L)	7.77 ± 1.64	7.4 ± 1.4	7.9 ± 1.7	0.444
Gamma globulin (g/L)	9.75 ± 3.80	8.1 ± 3.1	10.2 ± 3.9	0.096
A/G ratio	1.09 ± 0.22	0.98 ± 0.12	1.13 ± 0.23	0.033

*Data are presented as numbers (%), medians with interquartile range (IQR), and mean with standard deviation (SD). Statistical significance for group comparisons was tested using Mann-Whitney U test for quantitative variables presented as median (IQR) and Student’s *t* test for variables presented as mean±SD.

Arterial blood gases on the initial and second day of incident hospitalization showed no significant differences between the groups, although Paco_2_ was higher in the IMV group on the day of admission and showed a significantly greater fall on the second day, as expected (Table 3).

**Table 3:** Arterial blood gases on initial and following day according to the need for mechanical ventilation (N = 62)*

	Parameter values^†^			*P*
Parameter	total (N = 62)	mechanical ventilation (n = 14)	no mechanical ventilation (n = 48)	
pH on admission day	7.31 ± 0.09	7.30 ± 0.10	7.31 ± 0.08	0.594
HCO_3_ on admission day (mmol/L)	37.4 ± 8.6	40.7 ± 8.0	36.6 ± 8.7	0.127
Paco_2_ on admission day (mmHg)	74.6 ± 22.2	82.2 ± 21.6	73.3 ± 21.9	0.196
Pao_2_ on admission day (mmHg)	45.6 ± 19.4	51.4 ± 33.2	44.2 ± 13.9	0.979
Sao_2_ on admission day (%)	68.0 ± 18.6	65.8 ± 21.4	68.9 ± 18.3	0.615
Pao_2_/FiO_2_ on admission day	173.0 ± 52.7	178.5 ± 74.7	171.2 ± 44.0	0.880
pH on second day	7.35 ± 0.07	7.38 ± 0.09	7.34 ± 0.07	0.149
pH change	0.04 ± 0.10	0.08 ± 0.15	0.03 ± 0.08	0.054
HCO_3_ on second day (mmol/L)	36.8 ± 8.8	38.4 ± 9.8	36.5 ± 8.5	0.501
HCO_3_ change (mmol/L)	1 (-4 to 4)	0 (-5 to 2)	1 (-2 to 5)	0.504
Paco_2_ on second day (mmHg)	69.5 ± 19.8	67.1 ± 14.3	70.9 ± 20.9	0.535
Paco_2_ change (mmHg)	-2.5 (-15.5 to 5)	-5 (-24 to 1)	0.5 (-11 to 6)	0.032
Pao_2_ on second day (mm Hg)	69.4 ± 15.1	71.4 ± 15.4	68.9 ± 15.3	0.607
Pao_2_ change (mm Hg)	24.1 ± 23.9	20.8 ± 37.7	25.0 ± 19.2	0.609
Sao_2_ on second day (%)	90.3 ± 6.0	91.4 ± 5.1	89.9 ± 6.3	0.432
Sao_2_ change (%)	22.7 ± 18.9	25.7 ± 22.8	21.5 ± 17.9	0.482
Pao_2_/FiO_2_ on second day	220.8 ± 52.1	217.2 ± 47.2	221.8 ± 54.0	0.820

*Abbreviations: HCO_3_ – bicarbonate, Paco_2_ – partial pressure of carbon dioxide, Pao_2_ – partial pressure of oxygen, Sao_2_ - oxygen saturation, Pao_2_/FiO_2_ – ratio of arterial oxygen partial pressure to fractional inspired oxygen.

†Data are presented as medians with interquartile range (IQR) and means with standard deviation (SD). Statistical significance for group comparisons was tested using Mann-Whitney U test for quantitative variables presented as median (IQR) and Student’s *t* test for variables presented as mean±SD.

### Outcomes

Of 62 patients treated at ICU due to AECOPD, 7 (11.3%) died during incident hospitalization and 21 (33.9%) died during the follow-up (Table 1); thus, the overall 2.4-year mortality was 45.2%. A multivariate model showed that the survival time after incident hospitalization was independently associated with absolute number of monocytes, serum LDH level, comorbid cancer, hypertension, and BMI (chi^2^ = 40.080, df = 5, *P* < 0.001 for the overall model fit, Cox proportional hazard regression; Table 4).

**Table 4:** Significant predictors for the survival time after incident hospital admission (Cox proportional hazard regression analysis)*

Covariate	b	SE	Wald	*P*	Exp(b)	95% CI of Exp(b)
Monocyte count	0.584	0.204	8.209	0.004	1.792	1.202 to 2.671
Lactate dehydrogenase	0.007	0.003	6.674	0.010	1.007	1.002 to 1.012
Cancer	2.774	0.733	14.366	<0.001	16.020	3.817 to 67.239
Arterial hypertension	-1.736	0.727	5.710	0.017	0.176	0.042 to 0.732
BMI	-0.200	0.047	18.173	<0.001	0.818	0.746 to 0.897

*Abbreviations: SE – standard error, CI – confidence interval, BMI – body mass index.

Twenty nine (46.8%) patients had one or more readmissions after discharge. A multivariate model showed that time to readmission was independently associated with Paco_2_ on admission day, pH on the second day, levels of fibrinogen, proteins and alpha 2 globulins (chi^2^ = 20.161, df = 5, *P* = 0.001 for the overall model fit, Cox proportional hazard regression; Table 5). According to the multivariate model, age on admission, absolute number of neutrophils, no coronary artery disease (CAD), levels of sodium, and bilirubin were significantly associated with the number of readmissions during the median follow-up time of 2.4 years (Table 6).

**Table 5:** Significant predictors for the time to next hospitalization (Cox proportional hazard regression analysis)*

Covariate	b	SE	Wald	*P*	Exp(b)	95% CI of Exp(b)
Paco_2_ on admission day	0.031	0.015	4.099	0.042	1.031	1.001 to 1.062
pH on a second day	-11.090	3.988	7.735	0.005	0.000	6.15*10^−9^ to 0.038
Fibrinogen	-0.519	0.162	10.294	0.001	0.595	0.433 to 0.817
Total serum protein	-0.134	0.043	9.921	0.002	0.874	0.804 to 0.951
Alfa_2_ globulin	0.554	0.210	6.923	0.009	1.739	1.152 to 2.627

*Abbreviations: SE – standard error, CI – confidence interval.

**Table 6:** Significant predictors for the number of hospitalizations after the incident one*

Predictor	Estimate	Standard Error	Wald	95% CI	*P*
Intercept	-14.981	5.543	7.305	-25.844 to -4.117	0.007
Age at admission	-0.029	0.014	4.263	-0.056 to -0.001	0.039
Neutrophils	-0.202	0.056	12.929	-0.311 to -0.092	<0.001
No CAD	0.586	0.199	8.652	0.196 to 0.977	0.003
Sodium	0.137	0.039	12.111	0.060 to 0.213	<0.001
Bilirubin	-0.086	0.041	4.288	-0.166 to -0.005	0.038

*Abbreviations: CI – confidence interval, CAD – coronary artery disease.

The need for IMV during the incident hospitalization was present in 14 (22.6%) patients. Multivariate analysis revealed five independent variables as significant predictors for IMV during incident hospitalization. These variables included blood pH and bicarbonate levels, low albumin, low serum chloride, and low hemoglobin (Table 7). The model has an area under the curve (AUC) of 0.951 (95% confidence interval (CI), 0.856 to 0.991), sensitivity of 76.92% (95% CI, 49.74%-91.82%), specificity of 97.62% (95% CI, 87.68%-99.58%), positive predictive value (PPV) of 90.91% (95% CI, 62.26%-98.38%), negative predictive value (NPV) of 93.18% (95% CI, 81.77%-97.65%), and diagnostic accuracy of 92.73% (95% CI, 82.74%-97.14%) (chi^2^ = 29.609, df = 5, *P* < 0.001 for the model).

**Table 7:** Significant predictors for mechanical ventilation*

Variable	Coefficient	Standard error	Wald	*P*	Odds ratio	95% CI
pH	-14.137	6.900	4.197	0.041	0.001	10^−14^ to 0.54
HCO_3_	0.142	0.074	3.652	0.056	1.15	1.00 to 1.33
Low albumin	3.877	1.503	6.658	0.010	48.30	2.54 to 918.57
Low chloride	2.169	1.151	3.549	0.059	8.75	0.92 to 83.58
Low hemoglobin	3.759	1.464	6.592	0.010	42.91	2.43 to 756.37

*Abbreviations: CI – confidence interval, HCO_3_ – bicarbonate.

## DISCUSSION

We determined the predictors of poor outcomes in patients treated for severe AECOPD with respiratory failure in ICU of a tertiary respiratory medical center. As expected for such a high-risk group of patients, we found a high overall mortality, with an in-hospital mortality of 11.3% and post discharge mortality of 38.2% during an average follow-up period of 2.4 years. The possible independent predictors for overall survival, readmission risk, and respiratory failure with the necessity for IMV in our AECOPD patients with high mortality risk were hypoalbuminemia or anemia, which could be modifiable or treatable. Connors et al (13) reported similar in-hospital mortality and two-year mortality in patients hospitalized due to AECOPD and treated in open wards and ICU. Gunen et al (14) prospectively followed patients all hospitalized due to AECOPD for three years after discharge and reported similar percentages of overall in-hospital mortality, two-year mortality, and three-year mortality, but found that the subgroup of patients hospitalized in ICU had in-hospital mortality of 27%. Clinical characteristics of our patients regarding Paco_2_, pH, age, duration of COPD were worse than those in Gunen's group of patients (14), reflecting a more severe degree of respiratory failure and clinical condition. According to ICNARC Case Mix Program Database, 23.1% of patients who need treatment in ICU die during their stay in the ICU, and 38.8% during hospitalization (6). Results of that study showed lower average Paco_2_ and pH than those found in our patients, which could indicate acute onset of deterioration (8). Seneffe et al (15) found in-hospital mortality of 24% in patients treated in ICU after AECOPD. Mortality in our group of patients was comparable to the overall mortality data for AECOPD in hospitalized patients, but significantly lower than the mortality of patients treated in an ICU, possibly due to better care provided at the tertiary care ICU specialized for respiratory disorders.

Different factors are associated with survival after AECOPD. McGhan et al (6) showed that long-term survival after severe COPD exacerbations was associated with age, sex, prior hospitalizations, and comorbidities, including weight loss and pulmonary hypertension. In a study by Seneffe et al (15), variables associated with hospital mortality were age, weight, dysfunction of respiratory and other organs, and length of hospital stay before ICU admission, while respiratory physiological variables (respiratory rate, serum pH, Paco_2_, Pao_2_, and alveolar-arterial difference in partial pressure of oxygen) were associated with a 6-month mortality. Connors et al (13) reported that survival time was associated with disease severity, BMI, age, previous functional status, Pao_2_/FIO_2_, congestive heart failure, serum albumin, and cor pulmonale. In our patients, survival time was independently associated with monocyte count, LDH serum level, comorbidities including malignant disease and hypertension, and BMI.

Readmissions of patients hospitalized for AECOPD are frequent and occur in approximately 60% of patients in the first year after the incident hospitalization (16). AECOPD accelerates the decline of lung function, deteriorates quality of life and health status, and increases mortality (13,17,18). The strongest predictors of AECOPD recurrence are previous frequent exacerbations (18). Other identified risk factors include poor lung function, poor physical status (16), older age (19), poor quality of life (20), hypercapnia, and pulmonary hypertension (21). Our results are somewhat different, independently associating time to first readmission with Paco_2_ on admission, pH on the second day, and levels of fibrinogen, proteins, and alpha 2 globulins. The number of readmissions was independently associated with age, neutrophil count, CAD, and levels of sodium and bilirubin.

COPD patients often have associated diseases that significantly affect their management and prognosis (22). In our study, patients with AECOPD in need for IMV had significantly lower RBC counts, hemoglobin, hematocrit, bilirubin, albumin, and A/G ratio, and significantly increased troponin and alpha 1 globulin levels, but IMV was independently associated only with low albumin, low serum chloride, and low hemoglobin.

Anemia is often a comorbidity in COPD patients and its prevalence ranges from 12.3% up to 23%, being even higher in AECOPD (up to 50%), and is directly associated with negative outcomes and death (23-26). In our study, almost a third of patients had anemia, with more than half of them requiring IMV in comparison with 9.5% of those without anemia. In patients with severe COPD, anemia is associated with shorter survival, frequent hospitalizations, and longer hospital stay in comparison with patients without anemia (27). Anemic COPD patients have increased serum erythropoietin, indicating erythropoietin resistance that can be explained by inflammatory mechanisms (24). In a recent study in patients with AECOPD, C reactive protein, interleukin-6, tumor necrosis factor, and erythropoietin were found to be significantly higher at the time of hospitalization when compared with values at the time of AECOPD resolution (28).

Increased respiratory load associated with AECOPD increases the need for oxygen and aggravates anemia, further reducing muscle strength, worsening hypoxemia, with anemia further reducing oxygen delivery to periphery, all leading to early anaerobic metabolism ultimately resulting in respiratory collapse (29,30). Correction of anemia significantly reduces minute ventilation, respiratory load, and the possibility of weaning from IMV (27,31).

Lower concentrations of serum proteins have already been identified as predictors of poor outcome in AECOPD patients (14,32,33). Clinically significant threshold for mortality in the study of Gunen et al (14) was below 2.5 g/L. In our study only 3 (4.8%) patients had albumin below 2.5 g/dL and two of them were on IMV.

The prevalence of elevated troponin level in AECOPD is common, ranging from 18% up to 74% (34). Elevated troponin levels often indicate the existence of a cardiac injury in AECOPD patients, associated with higher mortality (34-36). The study by Noorain et al (37) showed that 38% of patients had elevated troponin I, which was closely associated with ICU admission and mechanical ventilation. There are several causes, including hypoxia, which can lead to cardiac injury associated with AECOPD (34). Increased left ventricle afterload due to negative intrathoracic pressure is one of them (38). During exacerbations of COPD, alveolar hypoxia and the consequent increase in pulmonary vascular resistance lead to a deterioration of pulmonary hypertension and overload of the right heart (38). This can also be the consequence of a pulmonary embolism, not a rare cause of clinical deterioration and breathlessness in COPD patients, often remaining unrecognized (39). Also, cardiovascular disease is a frequent comorbidity and a significant cause of morbidity and mortality in COPD patients (40-44). In patients with AECOPD and an elevated troponin I level, coronary angiography confirmed ischemic heart disease in 67%, and 38.6% required a coronary revascularization (42). In our patients, additional diagnostic evaluation (echocardiography, coronary angiography, and CT pulmonary angiography) was performed to exclude acute coronary incident or pulmonary embolism.

The limitations of our study are a relatively small number of patients and its retrospective design, which leave room for a possible bias. On the other hand, 95% CIs for all significant predictors of outcomes had narrow intervals well away from the margin of no effect. Also, as COPD patients have many comorbidities affecting survival, it would be important to analyze the causes of death. However, in our study only the date of death was available for all deceased due to the common practice that autopsy is not mandatory even for the patients who died in hospitals. As this is a retrospective study of associations, ie, level of evidence 4, our results may be useful for generating hypothesis and should be further corroborated by interventional studies focused on the modifiable risk factors for negative outcomes and death.

To summarize, we found that anemia, hypoalbuminemia and elevated troponin levels were risk factors for respiratory failure with the need for IMV in patients with severe AECOPD. Determination of simple parameters including hematologic parameters, albumin, and troponin, can identify a group of COPD patients with the highest risk for deterioration requiring more intensive monitoring and admission to the ICU. Identification of high-risk COPD patients at the beginning of hospital treatment may lead to an appropriate and timely treatment, which may have a significant effect on the outcome in these patients.
